# Identification and activity of inhibitors of the essential nematode-specific metalloprotease DPY-31

**DOI:** 10.1016/j.bmcl.2015.10.077

**Published:** 2015-12-15

**Authors:** David J. France, Gillian Stepek, Douglas R. Houston, Lewis Williams, Gillian McCormack, Malcolm D. Walkinshaw, Antony P. Page

**Affiliations:** a WestChem School of Chemistry, University of Glasgow, University Avenue, Glasgow G12 8QQ, UK; b Institute of Biodiversity, Animal Health & Comparative Medicine, University of Glasgow, Bearsden Road, Glasgow G61 1QH, UK; c Institute of Structural & Molecular Biology, University of Edinburgh, Mayfield Road, Edinburgh EH9 3JR, UK

**Keywords:** Nematode, Anthelmintic, Metalloprotease inhibitor, Docking, Peptidomimetic

## Abstract

Infection by parasitic nematodes is widespread in the developing world causing extensive morbidity and mortality. Furthermore, infection of animals is a global problem, with a substantial impact on food production. Here we identify small molecule inhibitors of a nematode-specific metalloprotease, DPY-31, using both known metalloprotease inhibitors and virtual screening. This strategy successfully identified several μM inhibitors of DPY-31 from both the human filarial nematode *Brugia malayi*, and the parasitic gastrointestinal nematode of sheep *Teladorsagia circumcincta*. Further studies using both free living and parasitic nematodes show that these inhibitors elicit the severe body morphology defect ‘Dumpy’ (Dpy; shorter and fatter), a predominantly non-viable phenotype consistent with mutants lacking the DPY-31 gene. Taken together, these results represent a start point in developing DPY-31 inhibition as a totally novel mechanism for treating infection by parasitic nematodes in humans and animals.

## Graphical abstract

**Figure:**
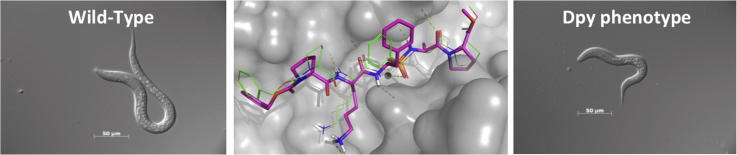

More than 1 billion people, predominantly in the developing world, are infected by parasitic nematodes (helminths). The primary strategy for eliminating these infections is preventive chemotherapy by mass anthelmintic drug administration, an approach that will select for drug resistance.^1^ Furthermore, helminth infection also represents a significant global burden to livestock.^2^ Resistance to anthelmintic drugs is increasing in gastrointestinal (GI) parasites of livestock, causing concern that this will also occur in human parasites. This increased resistance coupled with the limited availability of new drugs and absence of vaccines means that the identification of new potential targets for drug intervention is critical.^3^

The life cycle of all nematodes requires cyclical repetitive shedding of the organism’s protective cuticle and concomitant generation of a new cuticle at several points during maturation. This molting process involves a specific class of well-characterized astacin metalloproteases.^4^ The zinc endopeptidase DPY-31 is a nematode-specific member of this class that is essential for cuticle formation.4d A mutant suppressor screen in *Caenorhabditis elegans* identified the target of DPY-31 to be the cuticle collagen SQT-3, and specified the C-terminal cleavage domain of this crucial structural protein where DPY-31 acts.^5^ Critically, without the ability to molt, a nematode will fail to develop and ultimately die prematurely.

Here we describe the identification of small molecule inhibitors of DPY-31 employing two different approaches: use of known metalloprotease inhibitors and virtual screening. These compounds were screened for activity against recombinantly expressed DPY-31 from both the human filarial nematode *Brugia malayi*, and the parasitic GI nematode of sheep *Teladorsagia circumcincta*. Active compounds were then tested against both free living and parasitic nematodes themselves.

Seven known zinc protease inhibitors were included for testing (Fig. 1). The phosphinic pseudopeptide **1** used in the virtual screening described below is a mid-μM inhibitor of crayfish astacin and is studied here in the context of the nematode astacin DPY-31.^6^ Furthermore, the antibiotic and CD13/aminopeptidase N inhibitor actinonin (**2**),^7^ and the broad-spectrum matrix metalloprotease inhibitor marimastat (**3**),^8^ were examined. Four non-peptidic inhibitors of human procollagen C-proteinase (**4**–**7**) developed by Pfizer were also screened for in vitro activity against DPY-31.^9^

Finally, two tripeptide hydroxamic acids were prepared bearing two different carbamates on the N-terminus (Fig. 2). The hydroxamic acids were installed by CDI coupling of *O*-TMS-hydroxylamine with corresponding tripeptides, followed by hydrolysis.^10^ These structures were selected on the basis of studies showing the importance of a P1 aryl methyl group and P3 proline for binding to homologous crayfish astacin.^6^ It is also noteworthy that these *substrate* analogs are complementary to the *transition state* analog phosphinic pseudopeptide **1**.

A combination of ligand-based and structure-based methods were used for the in silico prediction of DPY-31 binding.^10^ Ligand-based screening was carried out by comparing a custom virtual library to molecules in the PDB that bind to enzymes homologous to DPY-31.^10^ Briefly, the custom virtual library was generated by merging the screening compound stock lists of several suppliers. The virtual library was then filtered according to the Oprea lead-like rules.^11^ This left 1,137,587 molecules, which formed the base library. A multiconformer version of this base library was produced using Multiconf-DOCK;^12^ resulting in a virtual library containing a total of 4,840,093 conformers. A search for compounds that contained one or more zinc-coordinating functional groups (hydroxamates, mercaptosulfides, phosphinic acids, sulfodiimines) was carried out using Sieve. 100 random conformations for each of the matches found were generated using Multiconf-DOCK. The programs UFSRAT and ROCS were used to search the custom virtual library for molecules with different types of similarity to the known ligands.^10^

As DPY-31 has not been crystallized, structure-based virtual screening was carried out using a 3D homology model of *C. elegans* DPY-31 constructed using Modeller^13^ (Fig.3A–C), and the structure of crayfish astacin in complex with phosphinic pseudopeptide transition state analog **1** (Fig. 1, PDB 1QJI).^6^ This resulted in a model with Modeller objective function of 1342.4 (Fig.3D).

The rigid-body docking program LIDAEUS was used to dock the conformer virtual library into the substrate binding groove of the DPY-31 model. The results were ranked and merged with the results from the ligand-based methods described above. These unique molecules were then docked into DPY-31 using Vina. The top compounds were then docked using Autodock and compounds whose predicted binding modes differed between the programs were discarded.^14^ Predicted binding poses were also scored using DrugScore 1.2.^15^ A final ranked list was prepared via a rank-by-rank consensus scheme,^16^ taking the Vina, Autodock, X-Score and DrugScore scores into account. The top 200 virtual hits were clustered according to similarity (Tanimoto < 0.7) and one compound from each cluster was selected for purchase (46 compounds). A further 28 compounds were selected for purchase as structural analogs of the molecules that were identified using the virtual screening techniques described above.

In total, 104 compounds were screened against recombinant DPY-31 from both the human parasite *B. malayi* as well as the sheep GI parasite *T. circumcincta* using an absorbance assay.(i), 10 Data for four of the most active compounds are given in Table 1. In keeping with the high level of sequence homology of DPY-31 across species,(g), (i) these inhibitors displayed broadly similar efficacy between the two species. Surprisingly, the phosphinic pseudopeptide **1** was inactive in this in vitro assay (IC_50_ > 500 μM). This may be due to the extremely slow binding kinetics of these inhibitors.^17^ Furthermore, shorter dipeptide hydroxamic acids (cf. **8** and **9**) were inactive in this assay.

Having successfully demonstrated small molecule inhibition of isolated DPY-31, we selected tripeptide hydroxamic acids **8** and **9** for phenotypic screening. These compounds were tested against three strains: free-living wild-type *C. elegans* N2, the *T. circumcincta dpy-31* transgenically-rescued *C. elegans dpy-31* mutant TP224, and parasitic *T. circumcincta* (Fig. 4). Phenotypes were evaluated in 96 well plate format over the course of 3 days using concentrations ranging from 50 μM to 2 mM. Both compounds were able to induce the Dpy phenotype that is consistent with loss of function of DPY-31.4d The similarity of these effects between wild-type *C. elegans*, the mutant strain, and *T. circumcincta* reinforces the conserved nature of this metalloprotease.

In conclusion, using a combination of in silico and experimental methods, we have identified small molecule inhibitors of the nematode-specific astacin metalloprotease DPY-31, which is essential for cuticle collagen biogenesis. These compounds are active against recombinant DPY-31 from both human and livestock nematode parasites. Furthermore, we have shown that these compounds can elicit the specific body morphology defect associated with deficiency of this essential protein in both free-living and parasitic nematodes. In *C. elegans*, these compounds replicate the phenotype associated with mutation of the *dpy-31* metalloprotease encoding gene.(d), (g) These results represent a first step toward validation of DPY-31 as a totally novel target for drug intervention in the treatment and control of parasitic nematodes of medical and veterinary significance. Future SAR work is expected to enhance potency while ensuring selectivity for DPY-31.

## Figures and Tables

**Figure 1:**
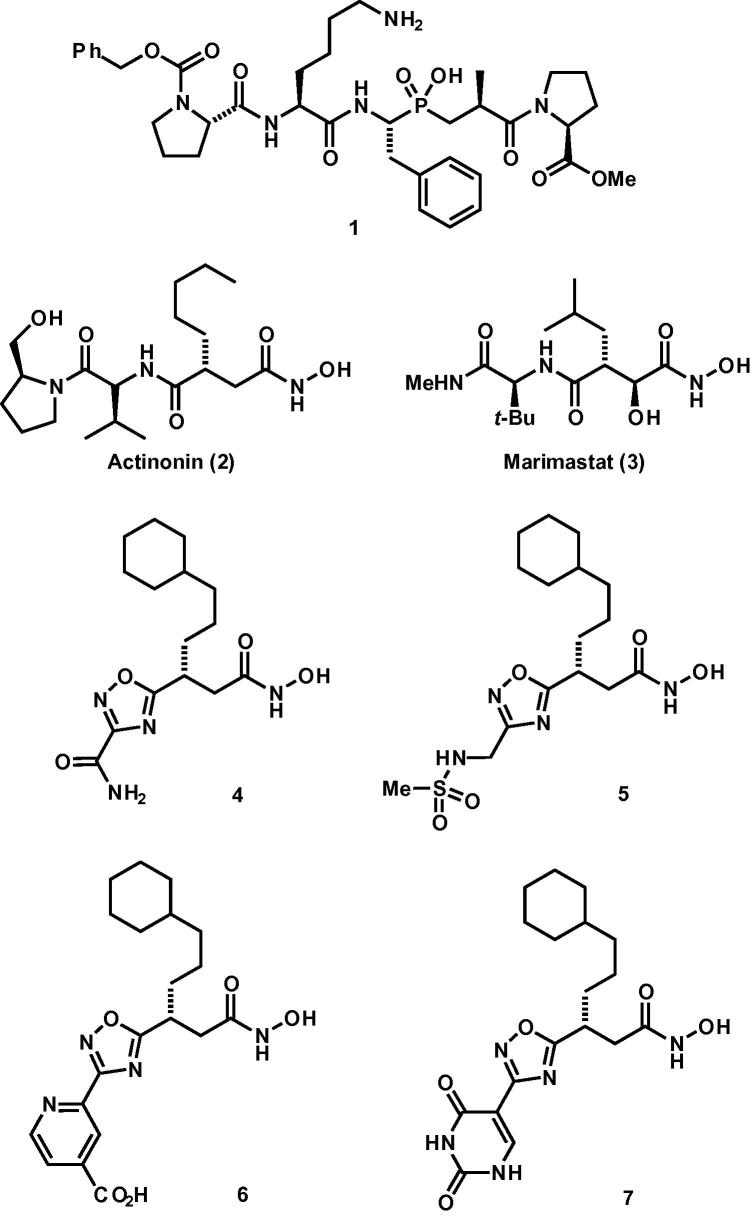
Known metalloprotease inhibitors screened against DPY-31.

**Figure 2:**
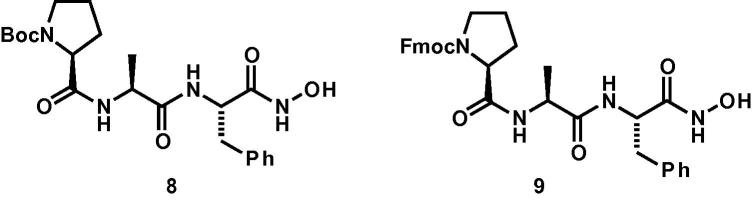
Novel tripeptide hydroxamic acids screened against DPY-31.

**Figure 3:**
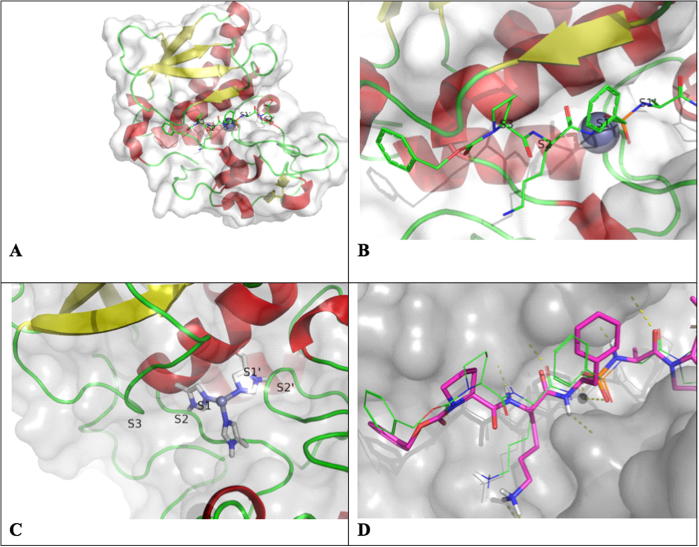
3D homology models of *C. elegans* DPY-31 alone, and crayfish astacin in complex with a phosphinic pseudopeptide transition state analog. (A) 3D homology model of *C. elegans* DPY-31, with (B) and (C) showing a closer view of the catalytic zinc-binding site, (D) 3D homology model of crayfish astacin in complex with phosphinic pseudopeptide transition state analog **1**.

**Figure 4:**
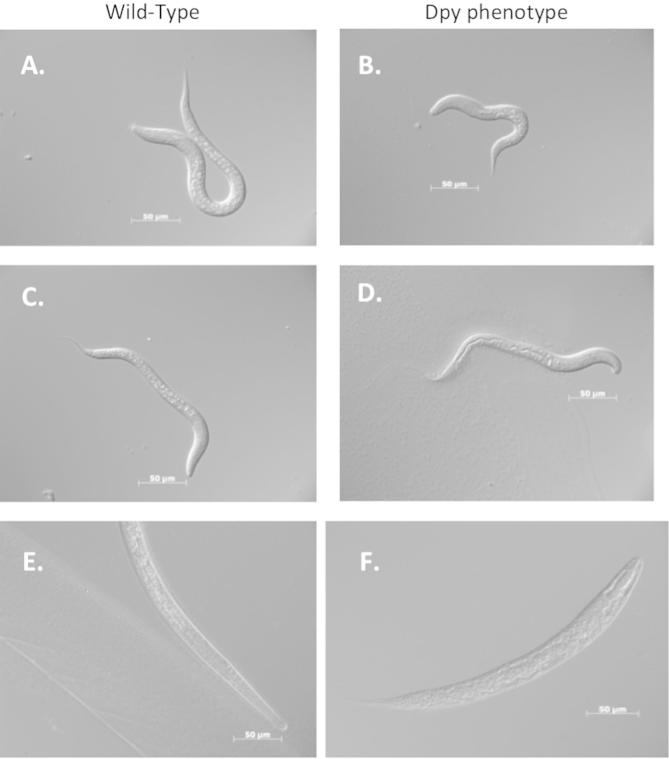
(a) WT L1 *C. elegans* (N2). (b) Dpy phenotype in L1 *C. elegans* (N2) with 50 μM **8**. (c) WT L1 transgenic rescue strain TP224. (d) Dpy L1 phenotype in TP224 with 100 μM **8**. (e) WT *T. circumcincta* L3. (f) Dpy phenotype in *T. circumcincta* L3 with 500 μM **9**.

**Table 1:** Inhibition of recombinant DPY-31 from *B. malayi* and *T. circumcincta* (±standard error)

Compound	pIC_50_ rDPY-31	
*B. malayi*	*T. circumcincta*
**3**	3.7 ± 0.2	4.1 ± 0.5
**6**	4.7 ± 0.3	4.4 ± 0.3
**8**	4.1 ± 0.4	4.6 ± 0.3
**9**	4.6 ± 0.4	4.5 ± 0.2

## Supplementary data

Supplementary data The in silico identification of potential astacin metalloprotease inhibitors, protocols for the recombinant expression of DPY-31, synthetic procedures and characterization for compounds **8** and **9**, and descriptions of the techniques used for absorbance assays and phenotypic screening. ^1^H and ^13^C NMR spectra for compounds **8** and **9**.
