# Supplementary material for: Identification and activity of inhibitors of the essential nematode-specific metalloprotease DPY-31
Source: Bioorg Med Chem Lett. 2015 Dec 15;25(24):5752–5. doi: 10.1016/j.bmcl.2015.10.077 (PMC4658336; doi:10.1016/j.bmcl.2015.10.077)
Supplement: Supplementary data — The in silico identification of potential astacin metalloprotease inhibitors, protocols for the recombinant expression of DPY-31, synthetic procedures and characterization for compounds 8 and 9, and descriptions of the techniques used for absorbance assays and phenotypic screening. 1H and 13C NMR spectra for compounds 8 and 9. [file mmc1.docx]

# Supplementary Material

1. *In silico* identification of potential astacin metalloprotease inhibitors

2. Recombinant expression of *B. malayi* and *T. circumcincta* DPY-31

3. Preparation of novel tripeptide hydroxamates

4. rDPY-31 Absorbance Assays

5. Phenotypic screening

6. References

7. NMRs

**1. *In silico* identification of potential astacin metalloprotease inhibitors**

*In silico* identification of compounds that are predicted to bind in the active site of *C. elegans* DPY-31 was performed as described below:

*1.1 Docking parameters and control experiments*

The template structure selected was Crayfish astacin in complex with a phosphinic pseudopeptide (PDB 1IQJ). This was selected on the basis of resolution, sequence identity, and the fact that almost all of the active site was occupied by the ligand, increasing the probability that the binding site residues were in ligand-binding conformations (as it is known that many proteases undergo some conformational change upon substrate binding). Fig S.I.-1 (below) shows the sequence alignment of the astacin domain of DPY-31 (NAS-35) and the sequence crystallised in PDB 1IQJ has an identity of 33.0% over 203 residues (the astacin domain). Water molecules were not included but the ligand HETATM records were. Special post-standard model building loop refinement protocols were not required as no loop regions were identified in the alignment. Global structural refinement was performed via the standard method included as part of the Modeller automodel.make() protocol (i.e. model optimisation via a Beale restart conjugate gradients algorithm in combination with the variable target function method, followed by model refinement by simulated annealing with molecular dynamics). Validation of the homology model was performed by the The Structure Analysis and Verification Server (version 4) metaserver (<http://services.mbi.ucla.edu/SAVES/>).

**Fig. S.I.-1.**


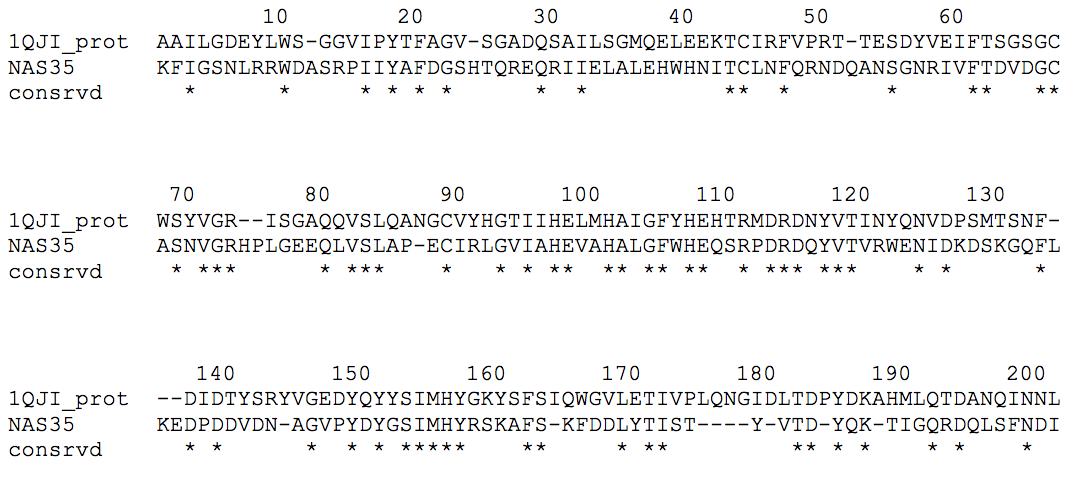


Water molecules and other hetero atoms were removed from the structures and the program PDB2PQR 1.8^[[1]](#endnote-1)^ was used to assign position-optimised hydrogen atoms, utilising the additional PropKa algorithm^[[2]](#endnote-2)^ with a pH of 7.4 to predict protonation states. The MGLTools 1.5.4 utility prepare_receptor4.py was used to assign Gasteiger charges to atoms, and MOPAC 2012^[[3]](#endnote-3)^ was used to calculate the charge of the zinc ion. Hydrogen atoms were assigned to ligand structures using OpenBabel 2.3.2,^[[4]](#endnote-4)^ utilising the -p option to predict the protonation states of functional groups at pH 7.4. The MGLTools utility prepare_ligand4.py was used to assign Gasteiger charges and rotatable bonds. As Vina 1.1.2^[[5]](#endnote-5)^ and Autodock 4.2.3^[[6]](#endnote-6)^ both use the same pdbqt format for their input, the same prepared files could be used for each. A grid box that encompassed the maximum dimensions of the ligand plus 12 Å in each direction was used. The starting translation and orientation of the ligand and the torsion angles of all rotatable bonds were set to random. The Autogrid grid point spacing was set at 0.2 Å. The Autodock parameter file specified 10 Lamarckian genetic algorithm runs, 2 000 000 energy evaluations and a population size of 300. Each docking program was used to automatically dock the phosphinic pseudopeptide ligand back into the substrate binding groove of the DPY-31 model.

*1.2 Curation of the virtual chemical library*

The screening compound stock lists in SDF format of ChemBridge, Asinex, Maybridge, Enamine, LifeChemicals, Specs, InterBioScreen, ChemDiv and KeyOrganics were merged. Salts were stripped out using Sieve 3.1.0 ([www.silicos-it.be](http://www.silicos-it.be)), and duplicates removed using canonical SMILES string comparison via OpenBabel 2.3.2. The supplied 2D coordinates were converted into 3D using Concord 4.08 (Pearlman RS, "Concord"; distributed by Tripos International, St. Louis, Missouri, 63144, USA). The virtual library was then filtered according to the Oprea lead-like rules (H-bond acceptors ≤ 9; H-bond donors ≤ 5; MW ≤460; cLogP ≥ -4.6 ≤ 4.2; cLogS ≥ -5; Number of rings ≤ 4; Number of rotatable bonds ≤ 9).^[[7]](#endnote-7)^ This left 1 137 587 molecules, which formed the base library.

A multiconformer version of the base library was produced using Multiconf-DOCK;^[[8]](#endnote-8)^ an average of 4.25 conformers per compound were generated depending on flexibility; this resulted in a virtual library containing a total of 4 840 093 conformers.

Compounds that contained one or more zinc coordinating functional groups (hydroxamates, mercaptosulphides, phosphinic acids, sulphodiimines) were searched for within the base library using Sieve. 100 random conformations for each of the matches found were generated using Multiconf-DOCK; this resulted in a custom virtual library containing 729 compounds represented by 57 339 conformers.

A second custom library was assembled by using the programs UFSRAT (Ultra Fast Shape Recognition with Atom Types),^[[9]](#endnote-9)^ ROCS (Rapid Overlay of Chemical Structures; ROCS, OpenEye Scientific Software;  Santa Fe, NM) and a Wiener index similarity comparison algorithm available as part of EDULISS^9^ to search the multiconformer version of the base library for molecules similar to those found in the PDB bound to enzymes with homology to DPY-31 (Table 1).

Table 1: Molecules in the Protein Data Bank (PDB) that bind to enzymes homologous to DPY-31

| **PDB molecules that bind to enzymes with homology to DPY-31** |
| --- |
| 1B3D 1B8Y 1BIW 1BM6 1BQO |
| 1C3I 1CAQ 1CIZ |
| 1D5J 1D7X 1D8F 1D8M |
| 1G05 1G49 1G4K |
| 1HY7 |
| 1QJI 1QJJ |
| 1SLN |
| 1UEA 1UMS 1UMT |
| 2JNP 2JT5 2JT6 |
| 2SRT |
| 3USN |

An expansion of the validated USR technique,^[[10]](#endnote-10)^ the UFSRAT similarity calculation process, consists of three steps: first, shape and atom property descriptors are calculated for each molecule; second, the descriptors are compared using a scoring function, and finally, similar molecules are ranked by score. The UFSRAT search of the multiconformer library returned 20 038 molecules.

ROCS is also a type of shape matching algorithm but uses a smooth Gaussian function to represent the molecular volume, analytically optimizing the volume intersection of the two molecules being compared.^[[11]](#endnote-11)^ The ROCS search of the multiconformer library returned 16 121 molecules.

Wiener index comparison is a well-known and long-established method in chemical graph theory that can be used to measure molecular similarity.^[[12]](#endnote-12)^ The calculation of this index by EDULISS 2.0 deduces the path number and the polarity number of a compound. The EDULISS search returned 10 702 molecules.

The merging of these results left 52 415 molecules after duplicates were removed.

*1.3 Ligand-based virtual screening (LBVS)*

The programs UFSRAT and ROCS were used to search the custom virtual library for molecules with different types of similarity to the known ligands. The UFSRAT search of the multiconformer custom virtual library returned 21 molecules. The ROCS search of the multiconformer custom virtual library returned 25 molecules. The results were merged, duplicates removed, and a total of 21 compounds were selected for acquisition.

*1.4 Structure-based virtual screening (SBVS) results*

The rigid-body docking program LIDAEUS was used to dock the conformer virtual library into the substrate binding groove of the DPY-31 model.^[[13]](#endnote-13)^ The results were ranked according to LIDAEUS score, the top 24 997 compounds from this list merged with the results from the ligand-based methods described above, and the duplicates removed. This resulted in 42 342 unique molecules which were then docked into DPY-31 using Vina. It is common in structure-based virtual screening to employ more than one docking algorithm in a consensus fashion as this is known to increase overall accuracy of prediction.^[[14]](#endnote-14)^ The fact that LIDAEUS, being a rigid-body docking algorithm, can dock many more molecules in a given time period than Vina, a flexible docking algorithm, means that a stepwise workflow that starts with LIDAEUS and places the slower Vina afterwards to triage compounds is the optimal arrangement; Autodock is the slowest and so is placed last, to create a "filter funnel" process. Docked poses were scored using both Vina’s internal scoring algorithm and X-Score 1.2;^[[15]](#endnote-15)^ these scores were used via a “rank-by-rank” consensus scheme^[[16]](#endnote-16)^ to create a ranked list. The top 8 895 compounds were then docked using Autodock. Predicted binding poses were also scored using DrugScore 1.2.^[[17]](#endnote-17)^ A final ranked list was prepared via a rank-by-rank scheme, taking the Vina, Autodock, X-Score and DrugScore scores into account. The top 200 hits were clustered according to similarity (Tanimoto < 0.7) and one compound from each cluster was selected for purchase (46 compounds).

**2. Recombinant expression of *B. malayi* and *T. circumcincta* DPY-31.**

The 684bp and 675bp sequences encoding the astacin domains of *T. circumcincta* DPY-31, and *B. malayi* DPY-31, were codon-optimised for *E. coli* by GeneArt (Life Technologies), and cloned into vector pMA-T. These plasmids were then inserted into the pET28a(+) vector, expression of the encoded proteins was induced and the resulting proteins were purified following the methods described previously.^[[18]](#endnote-18)^ Zinc metalloprotease assays were performed to determine the activity of the recombinant DPY-31 proteins from these nematode species, using the previously described method.^18^

## 3. Preparation of novel tripeptides hydroxamates

*General methods:*

Reactions were performed in glassware that had been dried in an oven (150 °C) prior to use. These reactions were carried out with the exclusion of air using an argon atmosphere. NMR spectra were recorded on a Bruker DPX-400 spectrometer (^1^H NMR at 400 MHz and ^13^C NMR at 100 MHz) or a Bruker DPX-500 spectrometer (^1^H NMR at 500 MHz and ^13^C NMR at 126 MHz). Chemical shifts are reported in ppm. ^1^H NMR spectra were recorded with DMSO-d^6^ as the solvent using residual DMSO-d^5^ (δ = 2.50) as internal standard, and for ^13^C NMR spectra the chemical shifts are reported relative to the central resonance of DMSO-d^6^ (δ = 39.52). Signals in NMR spectra are described as singlet (s), doublet (d), triplet (t), quartet (q), quintet (quint), septet (sept), multiplet (m), broad (br) or combination of these, which refers to the spin−spin coupling pattern observed. Spin−spin coupling constants reported are uncorrected. Two-dimensional (COSY, HSQC, HMBC, NOESY) NMR spectroscopy was used where appropriate to assist the assignment of signals in the ^1^H and ^13^C NMR spectra. IR spectra were obtained employing a Shimadzu FTIR-8400 instrument with a Golden Gate™ attachment that uses a type IIa diamond as a single reflection element so that the IR spectrum of the compound (solid or liquid) could be detected directly (thin layer). High resolution mass spectra were recorded under ESI conditions by the analytical services at the University of Glasgow. Reactions were monitored by thin layer chromatography (TLC) on Merck silica gel 60 covered aluminum sheets. TLC plates were developed under UV-light and/or with an acidic ethanolic anisaldehyde solution or a KMnO_4_-solution. All reagents were purchased from commercial suppliers and used without further purification.

**8:** To a solution of Boc-Pro-Ala-Phe-OH (0.43 g, 1.0 mmol) in THF (0.05 M) was added carbonyl diimidazole (CDI, 1.2 equiv.). The solution was stirred at rt for 1 h, before the addition of *O*-trimethylsilyl hydroxylamine (3.2 equiv.). The resultant mixture was stirred for 16 h, before quenching with MeOH. The solution was stirred for 1 h, and concentrated *in vacuo*. The crude mixture was diluted with water and acidified to pH 3 with 0.1 M aq. HCl. The mixture was extracted with CH_2_Cl_2_ (3 x 15 mL) and the combined organics were washed with sat. aq. NaHCO_3_ (3 x 15 mL), dried (Na_2_SO_4_), filtered and concentrated to yield the title compound (0.15 g, 0.33 mmol, 33%) as a white crystalline solid. ^1^H NMR (400 MHz, DMSO-*d*_6_) δ 10.60 (m, 1H), 8.90 (s, 1H), 8.01 – 7.88 (m, 2H), 7.26 – 7.17 (m, 5H), 4.37 – 4.06 (m, 3H), 3.27 – 3.16 (m, 2H), 2.93 – 2.77 (m, 2H), 2.08 – 2.00 (m, 1H), 1.79 – 1.71 (m, 3H), 1.37 – 1.29 (m, 9H), 1.18 – 1.13 (m, 3H); ^13^C NMR (126 MHz, DMSO-*d*_6_) δ 172.0, 171.6, 167.3, 153.2, 137.4, 129.1, 128.0, 126.3, 78.3, 59.2, 51.5, 47.9, 46.5, 37.9, 30.8, 28.0, 23.1, 18.3; IR (thin film) 1686, 1661, 1634, 1537, 1416; HRMS (ESI positive) exact mass calculated for C_22_H_32_N_4_O_6_Na [M+Na]^+^ m/z 471.2209; found m/z 471.2214.

**9:** To a solution of Fluorenylmethyloxycarbonyl (Fmoc)-Pro-Ala-Phe-OH (0.40 g, 0.75 mmol) in THF (0.05 M) was added carbonyl diimidazole (CDI, 1.2 equiv.). The solution was stirred at rt for 1 h, before the addition of *O*-trimethylsilyl hydroxylamine (3.2 equiv.). The resultant mixture was stirred for 16 h, before the quenching with MeOH. The solution was stirred for 1 h, and concentrated *in vacuo*. The crude mixture was diluted with water and acidified to pH 3 with 0.1 M aq. HCl. The mixture was extracted with CH_2_Cl_2_ (3 x 10 mL) and the combined organics were washed with sat. aq. NaHCO_3_ (3 x 10 mL), dried (Na_2_SO_4_), filtered and concentrated to yield the title compound (0.12 g, 0.21 mmol, 28 %) as a white crystalline solid. ^1^H NMR (400 MHz, DMSO-*d*_6_) δ 10.63 – 10.55 (m, 1H), 9.55 (1H, s), 8.27 – 7.82 (m, 4H), 7.71 – 7.58 (m, 2H), 7.48 – 7.13 (m, 9H), 4.44 – 4.07 (m, 6H), 3.49 – 3.40 (m, 2H), 3.09 – 3.01 (m, 1H), 2.96 – 2.86 (m, 1H), 2.25 – 2.01 (m, 1H), 1.96 – 1.57 (m, 3H), 1.20 – 1.09 (m, 3H); ^13^C NMR (126 MHz, DMSO-*d*_6_) δ 171.9, 171.7, 171.4, 154.0, 140.7, 140.6, 137.3, 129.2, 128.9, 128.2, 128.0, 127.6, 127.1, 125.3, 67.0, 66.5, 59.0, 54.9, 48.2, 46.7, 36.7, 31.3, 21.0, 18.2; IR (thin film) 1732, 1697, 1668, 1649, 1433; HRMS (ESI positive) exact mass calculated for C_32_H_34_N_4_O_6_Na [M+Na]^+^ m/z 593.2366; found m/z 593.2371.

**4. rDPY-31 Absorbance Assays**

An astacin assay was performed to determine the inhibitory activity of all compounds against recombinant DPY-31 from the two nematode species. Briefly, 150 μg/ml of recombinant enzyme was incubated with 0.1 mM ZnCl_2_ and various concentrations (5 μM to 400 μM) of compound, and the volume was made up to a total of 100 μl with 50 mM NaPO_4_, pH 8.0 buffer. Pre-incubation occurred for 3 hrs at 37 ^o^C prior to the addition of the substrate, 1 mM Suc-Ala-Ala-Ala-pNA (Bachem), and subsequent incubation at 37 ^o^C for a further 3 hr. Absorbance was measured on a plate reader at 405 nm. Each sample was performed in duplicate.

**5. Phenotypic screening**

*5.1 Screening of compounds against C. elegans N2 larvae*

Compounds **8** and **9** were screened against *C. elegans* N2 L4s using 96-well plates. A single L4 was placed into each well in a total volume of 100 μL, made up of compound, M9 buffer and 5 μl 10x concentrated food (*E. coli* strain OP50), and incubated at 20 ^o^C for 3 days, with the effects on the phenotypes recorded daily. The compounds were screened in duplicate at concentrations ranging from 50 μM to 2 mM. Actinonin (100 μM) was used as a positive control.^18^ Representative offspring were transferred onto 2% agarose/0.01% azide pads on slides and viewed with a Zeiss Axioskop2 microscope. Images were taken using an AxioCam camera and Axiovision software.

*5.2 Screening of compounds against the transgenic* T. circumcincta dpy-31 *rescued* C. elegans dpy-31 *mutant strain TP224*

Compounds **8** and **9** were screened against *C. elegans* N2 or TP224 L4s at 100, 250 and 1000 μM, using 96-well plates. A single L4 was placed into each of the wells in a total volume of 100 μl, made up of compound, M9 buffer and 5 μl 10x concentrated OP50. The compound concentrations were tested in duplicate for each compound. The plates were sealed with gas-permeable covers and incubated at 20 ^o^C for 3 days, with the effects on the phenotypes recorded daily. Representative worm images were taken as described in section 5.1.

*5.3 Screening of compounds against* T. circumcincta *L1s and L3s*

*T. circumcincta* L1s or exsheathed L3s were incubated with compounds **8** and **9** at 100, 500 and 1000 μM. 10-20 L1s were incubated per well of a 96-well plate, each well containing compound, 5 μl 10x concentrated OP50 and EBSS (Earle’s Balanced Salt Solution; Life Technologies) to 100 μl, and 10-20 L3s were incubated per well of a 96-well plate, each well containing compound and EBSS to 100 μl. Each compound was tested in duplicate for all concentrations. The plates were sealed with gas-permeable covers and incubated at 26^o^C (L1s) or 40^o^C (L3s) at 100 rpm for 7-8 days and any observations on development were recorded. Representative worm images were taken as described in section 5.1.

**6. References**

1. Dolinsky et al. (2007) Dolinsky TJ, Czodrowski P, Li H, Nielsen JE, Jensen JH, Klebe G, Baker NA. PDB2PQR: expanding and upgrading automated preparation of biomolecular structures for molecular simulations. Nucleic Acids Research. 2007;35:W522–W525. doi: 10.1093/nar/gkm276 [↑](#endnote-ref-1)
2. Li H, Robertson AD, Jensen JH Very fast empirical prediction and rationalization of protein pKa values. Proteins. 2005;61(4):704–21 [↑](#endnote-ref-2)
3. Stewart JJP. 2012. MOPAC2012. Colorado Springs, CO: Stewart Computational Chemistry. [↑](#endnote-ref-3)
4. O’Boyle, M.N., Banck, M., James, C.A., Morley, C., Vandermeersch, T., Hutchison, G.R. et al. 2011. Open Babel: An open chemical toolbox. J. Cheminf. 3:33. [↑](#endnote-ref-4)
5. Trott O, Olson AJ. AutoDock Vina: improving the speed and accuracy of docking with a new scoring function, efficient optimization, and multithreading. Journal of computational chemistry. 2010;31:455–461 [↑](#endnote-ref-5)
6. Huey R, Morris GM, Olson AJ, Goodsell DS A semiempirical free energy force field with charge-based desolvation. J Comput Chem. 2007;28(6):1145–52 [↑](#endnote-ref-6)
7. Hann MM, Oprea TI Pursuing the leadlikeness concept in pharmaceutical research. Curr Opin Chem Biol. 2004;8(3):255–63 15183323 [↑](#endnote-ref-7)
8. Sauton N, Lagorce D, Villoutreix BO, Miteva MA MS-DOCK: accurate multiple conformation generator and rigid docking protocol for multi-step virtual ligand screening. BMC Bioinformatics. 2008;9:184 [↑](#endnote-ref-8)
9. Hsin K-Y, Morgan HP, Shave SR, Hinton AC, Taylor P, Walkinshaw MD EDULISS: a small-molecule database with data-mining and pharmacophore searching capabilities. Nucleic Acids Res. 2011;39(Database issue):1042–8 [↑](#endnote-ref-9)
10. Patil SP, Ballester PJ, Kerezsi CR Prospective virtual screening for novel p53-MDM2 inhibitors using ultrafast shape recognition. J Comput Aided Mol Des. 2014;28(2):89–97 [↑](#endnote-ref-10)
11. Grant JA, Gallardo MA, Pickup B A fast method of molecular shape comparison: A simple application of a Gaussian description of molecular shape. Journal of Computational Chemistry. 1996;17:1653–66 [↑](#endnote-ref-11)
12. Rouvray DH The rich legacy of half a century of the Wiener index In: Rouvray DH, King RB, editors. Topology in Chemistry: Discrete Mathematics of Molecules: Horwood Publishing; 2002. p. 16–37 [↑](#endnote-ref-12)
13. Taylor P, Blackburn E, Sheng YG, Harding S, Hsin KY, Kan D, et al. Ligand discovery and virtual screening using the program LIDAEUS. Br J Pharmacol. 2008; 153 Suppl 1:S55–67 [↑](#endnote-ref-13)
14. See also: Chapter 4: Virtual Screening Triage. Schneider, G.; Baringhaus, K.-H.; Kubinyi, H. "Molecular Design: Concepts and Applications", Wiley-VCH Publishing, pp.149-189 [↑](#endnote-ref-14)
15. Wang R, Lai L, Wang S Further development and validation of empirical scoring functions for structure-based binding affinity prediction. J Comput Aided Mol Des. 2002;16(1):11–26 [↑](#endnote-ref-15)
16. Wang R, Wang S How does consensus scoring work for virtual library screening? An idealized computer experiment. J Chem Inf Comput Sci. 2001;41(5):1422–6 [↑](#endnote-ref-16)
17. Gohlke H, Hendlich M, Klebe G Knowledge-based scoring function to predict protein-ligand interactions. J Mol Biol. 2000;295(2):337–56 [↑](#endnote-ref-17)
18. Stepek, G., McCormack, G., Winter, A. D., Page, A.P., 2015. A highly conserved, inhibitable astacin metalloprotease from Teladorsagia circumcincta is required for cuticle formation and nematode development. Int. J. Parasitol. 45, 345-355.

    **7. NMR data for 8 and 9.**


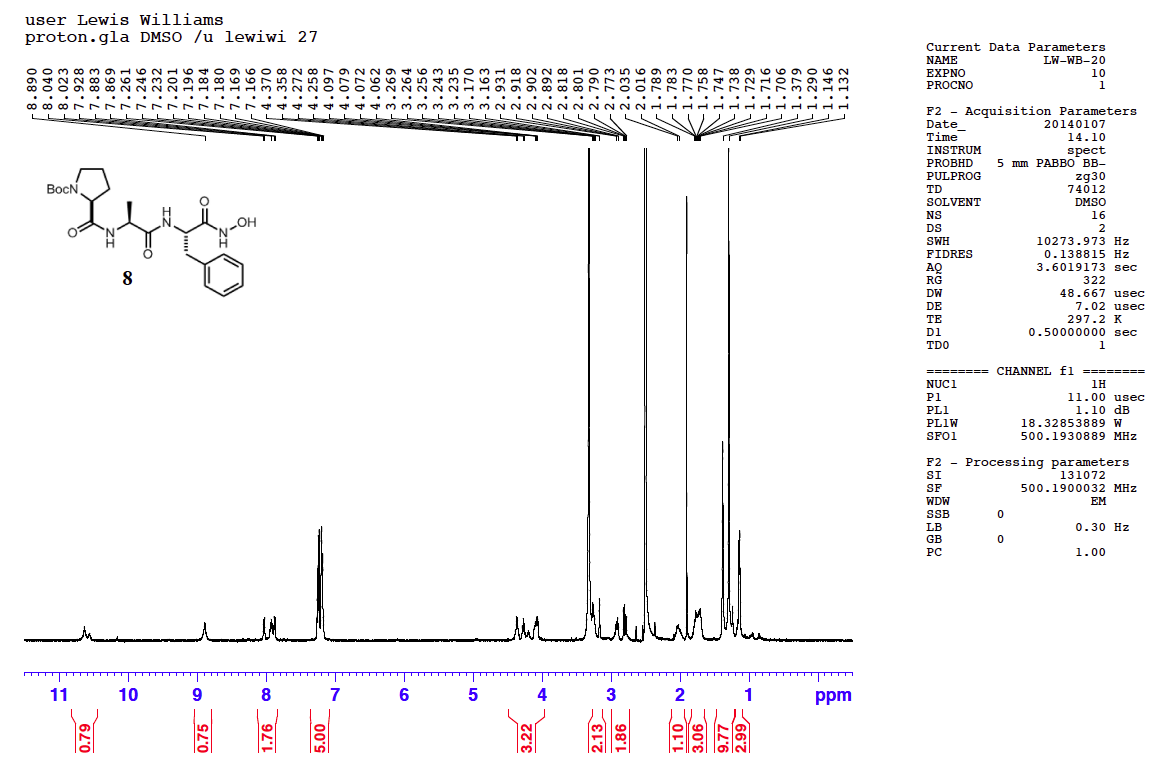


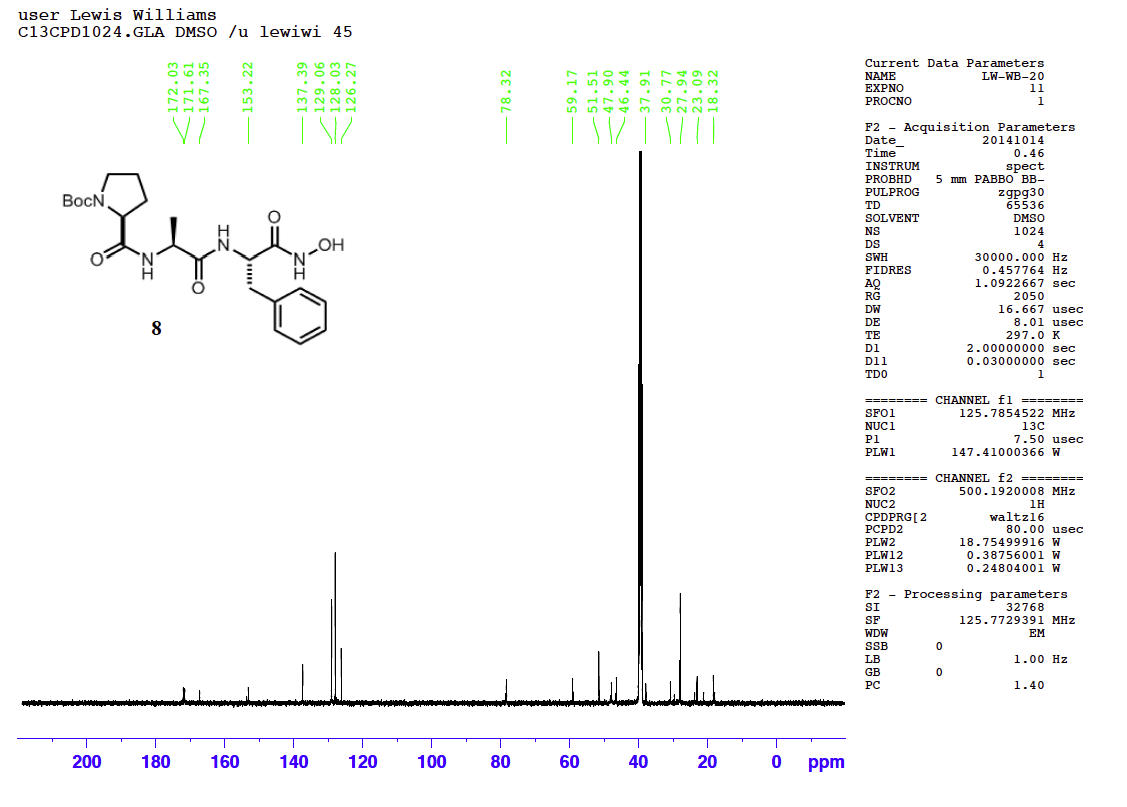


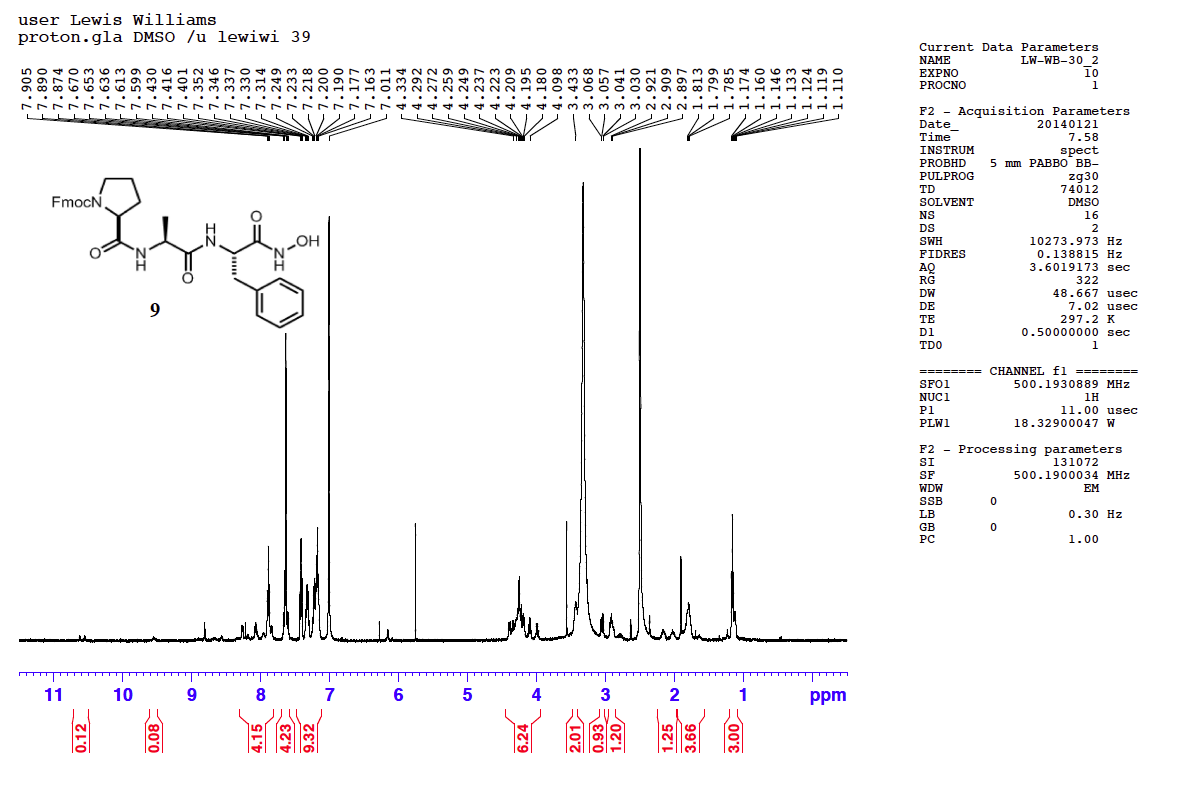


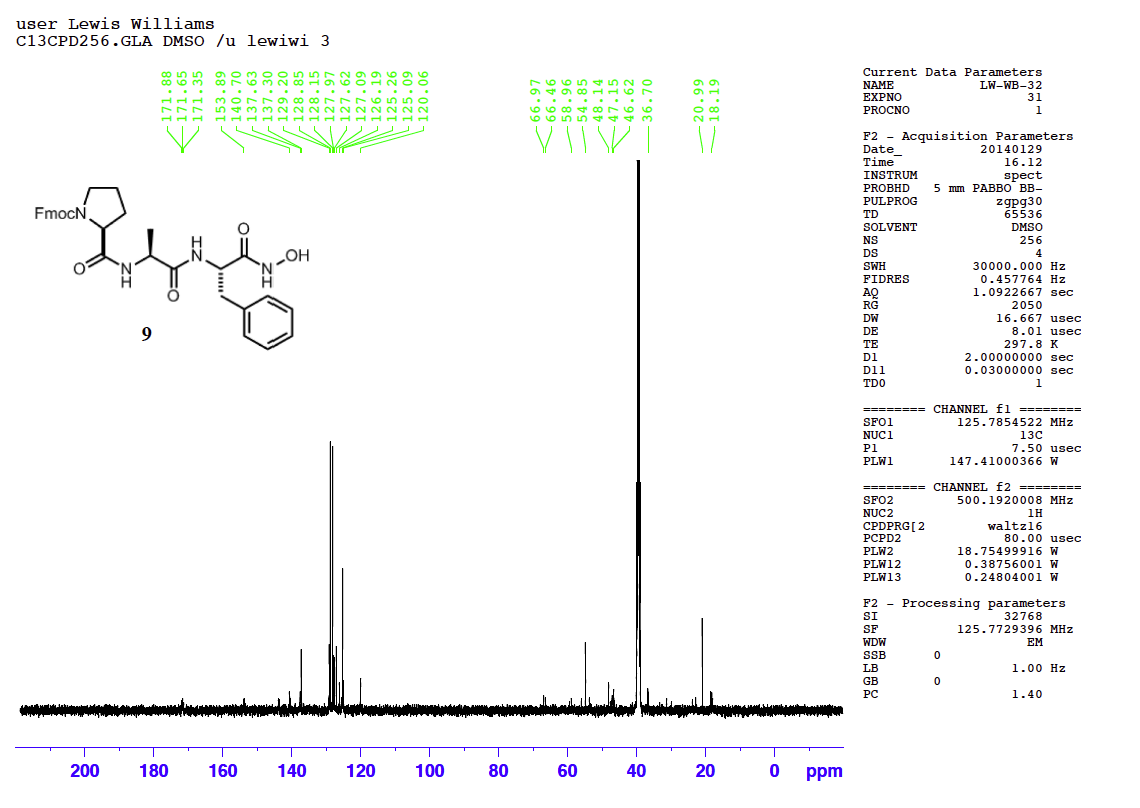
 [↑](#endnote-ref-18)
